# Local treatment in unresectable hepatic metastases of carcinoid tumors: Experiences with hepatic artery embolization and radiofrequency ablation

**DOI:** 10.1186/1477-7819-3-75

**Published:** 2005-11-17

**Authors:** Vincent Meij, Johanna M Zuetenhorst, Richard van Hillegersberg, Robert Kröger, Warner Prevoo, Frits van Coevorden, Babs G Taal

**Affiliations:** 1 Department of Surgery, Netherlands Cancer Institute/ Antoni van Leeuwenhoek Hospital, Amsterdam, The Netherlands; 2 Department of Medical Oncology, Netherlands Cancer Institute, Plesmanlaan 121, 1066 CX Amsterdam, The Netherlands; 3 Department of Radiology, Netherlands Cancer Institute/ Antoni van Leeuwenhoek Hospital, Amsterdam, The Netherlands

## Abstract

**Background:**

Hepatic metastases of carcinoid tumors cause incapacitating symptoms, but are usually diffuse and therefore unresectable. In this article we evaluate our experiences with local treatment techniques in the management of carcinoid patients with hepatic metastases and failing systemic treatment.

**Methods:**

Fifteen consecutive carcinoid patients (11 men and 4 women; median age 60 years; range 45–71 years) were treated with either hepatic artery embolization (HAE) with Ivalon particles or radiofrequency ablation (RFA) (percutaneously or intra-operatively). Follow-up evaluation was performed by CT scan and 24-hours urinary 5-HIAA excretions.

**Results:**

A total of 18 HAE's was performed in 13 patients, while 10 lesions in 3 patients were treated with RFA. Median follow-up was 12.5 months (2 – 25 months). Median duration of symptoms was 22 months (8 – 193 months). Median overall decrease of 5-HIAA excretion 2 months after HAE was 32% with tumor regression on CT-scan in 4 patients (30%) and improvement of symptoms with a median duration of 15 months in 3 of them (23%). Embolization led to fatal hepatic failure in one patient. The 3 patients treated with RFA showed a decrease of urinary 5-HIAA values of 34, 81 and 93% respectively, with tumor regression in all of them. Improvement of symptoms was reported in 2 patients up to 25 months.

**Conclusion:**

Liver embolization performed late in the clinical course had limited effect on symptoms and biochemical and radiological parameters. First experiences with RFA are favorable and might encourage to apply RFA more widely in metastatic carcinoid.

## Background

Carcinoid tumors are derived from neuroendocrine cells and are usually slowly growing malignant tumors with distinct morphological characteristics. Most frequently, carcinoids are located in the appendix and ileum, but they are also known to arise from other sites, such as the bronchus and pancreas. The carcinoid syndrome is mostly due to hepatic metastases with the release of hormones, such as serotonin, directly into the systemic circulation and is characterized by symptoms of flushing, diarrhea, and wheezing. Eventually right-sided heart-valve fibrosis may develop.

There are several treatment options in metastatic disease. A multidisciplinary approach is advocated, in which gastroenterologists, medical oncologists and surgeons all have a role [1]. Systemic treatment is aiming at symptomatic improvement and reduction of hormonal secretion. Somatostatin analogues, interferon-alpha and application of meta-iodobenzylguanidin (MIBG), pharmacological or combined with radioactive MIBG, can result in long-lasting palliation [2-6]. However, tumor reduction is only occasionally described with these treatment modalities and as tumor load increases, symptomatic treatment gradually fails.

Local treatment of hepatic metastases of carcinoid is attractive because of the slow and localized growth pattern. When metastases are restricted to one lobe of the liver a hemihepatectomy or segmental resection is advocated. Unfortunately, the metastases are often multiple and diffuse and therefore resection is usually impossible due to anatomical location or number or due to inadequate viable liver tissue that would remain after surgery. If feasible, hepatic resection can be performed safely and provides effective palliation with considerable duration [7].

Another local treatment option is hepatic artery embolization (HAE), which not only may ameliorate symptoms but also might reduce tumor burden. An objective biochemical response of up to 52% and a median duration of effect of 12 months have been reported in cases of failing systemic therapy [8]. Reports on chemoembolization show a slightly better biochemical response and tumor response [9].

Although radio frequency ablation (RFA) is a fairly new technique, various studies have demonstrated its effectiveness in the treatment of unresectable hepatocellular carcinomas and hepatic metastases of colon carcinoma [10,11]. In patients with diffuse hepatic metastasis RFA could achieve local tumor control while remaining enough functional liver tissue. Previous reports on RFA of hepatic carcinoid metastases are limited to case reports and small series but have indicated good local tumor control and acceptable morbidity [8,12-15].

Besides our experience in HAE, we have recently introduced the use of RFA in carcinoid patients. In this article we describe our experience of local treatment techniques in our institutional series of unresectable hepatic metastases of carcinoids and failing systemic therapy.

## Patients and methods

### Patients

Among 46 patients with carcinoid syndrome treated in the Netherlands Cancer Institute/ Antoni van Leeuwenhoek Hospital between January 2000 and June 2002 we evaluated the data of all patients (n = 15) who were treated with either hepatic artery embolization or RFA.

Indications were progressive disease with systemic treatment and multiple hepatic metastases not amenable for resection, because of diffuse localization and bilobar involvement. Somatostatine analogues, interpheron-alpha and meta-iodobenzylguanidin, pharmacological or combined with radioactive MIBG could no longer ameliorate symptoms in these patients. Treatment with RFA was selected in patients where this was technically possible and all metastases could be treated with sufficient remaining functional liver tissue and with safe margins to the portal vein. Before treatment CT scans were performed and urinary 5-hydroxyindole acetic acid (5-HIAA) excretion was measured.

### Embolization

The radiographic procedure was started with a diagnostic arteriography of the celiac axis and the superior mesenteric artery using the Seldinger technique to assess arterial anatomy. The portal vein patency was assessed on pre-embolization CT scan. Thereafter selective catheterisation was performed of the arterial supply of the right (segment 5–8) or left (segment 2–4) lobe pending on localization of the largest metastatic area. Ivalon particles 150–600 μ in polyvinyl alcohol mixed with an iodined contrast medium were injected until stasis was obtained. When tumor was present in both lobes of the liver, both lobes were embolized with an interval of several months starting with the lobe with the largest tumor load. In these cases, evaluation of symptoms and biochemical and radiological signs was assessed after the second embolization. After treatment vital signs, as well as hepatic and renal functions were monitored on the ward regularly. An adequate diuresis was ensured by careful hydration to avoid hepatorenal failure in the post-embolization period

### Radiofrequency ablation

RFA was performed either intraoperatively or percutaneously. A 3.5 or 3.0 LeVeen Needle was used in combination with a 90 watts radio wave generator (Radiotherapeutics, USA). After ultrasound-guided insertion of the needle, sequential increase per minute of the radio wave current was performed until a maximum of 90 watts was achieved. An increase in resistance ultimately resulted in a "roll off", after which a second procedure at the same localization was restarted after a one-minute interval without current, to a maximum of 70% of the first achieved level. Each localization was thus treated, or a combination of multiple passes of the LeVeen needle was used to cover a metastasis larger than 4 cm until complete tumor necrosis was achieved.

During follow-up symptoms and measurement of urinary 5-HIAA's were evaluated at least every 3 months in our outpatient clinic. Tumor size and necrosis of tumor were evaluated by a CT scan at 3, 6 and 12 months after treatment. In the patients who were treated with RFA an additional CT scan one month after the procedure was performed.

## Results

Eighteen hepatic artery embolizations were performed in 13 patients and 10 metastases were treated with RFA in 3 patients (table 1). There were 11 men and 4 women with a median age of 60 years (range 45 to 71) at time of treatment. The primary tumor was located in the ileocoecal area in 7 patients, rectum in 1 patient, lung in 3 patients, while in the remaining 4 patients the primary tumor was unknown.

**Table 1:** Characteristics of patients

**No.**	**Age at local therapy**	**Duration of symptoms (months)**	**Gender**	**Primary tumour**	**Local therapy**	**Location of therapy**
1	45	78	F	unknown	emb.	right
2	67	24	M	unknown	emb.	right
3	53	11	M	rectum	emb.	bilat.
4	71	67	M	lung	emb.	right
5	70	63	F	lung	emb.	right
6	60	26	M	ileocoecal	emb.	bilat.
7	62	24	M	appendix	emb.	left
8	60	11	F	ileocoecal	emb.	right
9	61	23	M	lung	emb.	right
10	71	10	M	unknown	emb.	bilat.
11	52	21	M	ileocoecal	emb.	bilat.
12	59	16	M	unknown	emb.	bilat.
13a	55	11	M	ileocoecal	emb.	right
13b	55	17	M	ileocoecal	RFA	left
14	47	8	F	ileocoecal	RFA	bilat.
15	60	193	M	ileum	RFA	bilat.

Indications for RFA were a vascular anomaly because of which HAE could not be performed in 1 patient. The other patients treated with RFA had very large lesions among several smaller ones, which made them more appropriate for RFA. The diagnosis of metastatic disease was known during a considerable time before local treatment (median duration of symptoms: 22 months, range: 8–193 months). As shown in table 2, 14 of the 15 patients had elevated urinary 5-HIAA excretion values with a median of 913 μmol/24 hrs (range 35 – 2618 μmol/24 hrs) (normal <40 μmol/24 hrs) prior to embolization. Median follow-up from completion of therapy was 12,5 months (range 6 – 25 months). Seven patients were dead at the time of analysis.

**Table 2:** Biochemical and radiological effects and effects on symptoms

**No.**	**5-HIAA before**	**5-HIAA after 2 months**	**5-HIAA at follow-up**	**Effect on tumoursize on CT-scan**	**Effect on symptoms**	**Follow-up (months)**	**Complications**
1	1736	unknown	unknown	unknown	=	2(†)	no
2	35	23	23	progression	↓	4(†)	postembolization syndrome
3	120	160	42	progression	=	7(†)	hepatic failure
4	1924	1549	1432	progression	=	10(†)	no
5	111	75	123	progression (after 9 months)	=	11(†)	postembolization syndrome
6	1662	1564	2022	progression	=	12(†)	no
7	1740	1617	1604	progression (after 5 m)	=	14	abscess
8	913	140	566	regression	↑	14	postembolization syndrome
9	1961	1531	2050	no change	=	14(†)	postembolization syndrome
10	72	33	118	regression	↑	15	no
11	875	720	765	progression	=	15	no
12	1529	165	502	regression	↑↑	22	postembolization syndrome
13a	2618	1458	1186	regression	=	6*	no
13b	1186	777	1815	regression	=	7	no
14	91	6	32	regression	↑	13	cholestasis
15	277	52	79	regression	↑	25	no

(↓: worsening of symptoms, =: no change, ↑: improvement of symptoms, ↑↑: free of symptoms).

Embolization resulted in an overall median decrease of 5-HIAA excretion of 32% (range 0–89%) at first follow-up. In 3 patients, (23%; 95%-confidence interval: 5–54%) the decrease after embolization was more than 50% and met the criteria of biochemical response (patient 8, 10 and 12). In these 3 patients, symptoms improved, including patient 12 who were free of symptoms after bilateral embolization. This symptomatic improvement in the patients with biochemical response was still present at follow-up of 14, 15 and 22 months respectively. In the remaining ten patients only a minimal biochemical response was achieved with no effect on symptoms.

Tumor regression on CT scan was present in 4 patients, which corresponded with symptomatic improvement in three of them. In 7 patients CT-scans revealed progression and in one there was no change in tumor size. In one patient radiological evaluation could not be performed as this patient died after two months because of cardiac failure.

Postembolization syndrome, characterized by elevated levels of transaminases, lactate dehydrogenase and alkaline phosphatase combined with fever, occurred in 5 patients. All recovered within 10 days. More serious complications occurred in 2 cases. In one patient (nr.7) a necrotic tumor metastasis was infected, for which treatment with intravenous antibiotics and abscess drainage was needed. After embolization of the left artery in another patient (nr.3), embolization of the right hepatic artery was performed after 5 months, which led to fatal hepatic failure 2 months later. Six other patients died during follow-up because of non-intervention related problems after 2 to 14 months: right sided cardiac failure (n = 2), progressive disease (n = 2), carcinoid crisis with renal failure (n = 1) and pneumonia (n = 1).

Treatment with RFA induced a biochemical response in 2 out of 3 patients with a 5-HIAA reduction of 81% and 93%. They also experienced a radiological response, and a symptomatic improvement. Patient 14 underwent RFA for one large and several smaller lesions. Patient 15 underwent a resection of segment 6 and 7, because of a large lesion of 7 cm in diameter and RFA of 5 other lesions. In the patient with the longest follow-up the decrease in 5-HIAA excretion after 26 months was still 71% and symptoms were minimal without additional systemic treatment.

In patient 13 with a favorable response to embolization of the right hepatic artery, embolization of the left hepatic artery was technically impossible because of a vascular anomaly. He was subsequently treated with multiple passes of RFA percutaneously for two large lesions (6 and 7 cm in diameter) in the let lobe under spinal anesthesia leading to a further reduction in 5-HIAA excretion. However, although CT scans after RFA showed no recurrence of tumor, duration of decreased 5-HIAA values lasted only 3 months and a relief of symptoms could not be obtained.

No serious adverse effects occurred after RFA. In one patient (nr.14) post-operative cholestasis was present. A magnetic resonance cholangiopancreatography revealed a minor stenosis of the common bile duct. Symptoms completely recovered after 2 months and hepatic values returned to normal values after 3 months, without additional treatment.

## Discussion

In carcinoid tumors with liver metastases patients suffer from the production of vasoactive hormones. Various systemic treatments are available to diminish the incapacitating symptoms of the carcinoid syndrome. Somatostatin analogues are widely applied and reduce these symptoms in approximately 70%, while a biochemical response is achieved in 42% up to 72% and a duration of up to a year [2,6]. Interferon-alpha has demonstrated similar effects [3]. Application of meta-iodobenzylguanidin (MIBG), in a pharmacological dosage or combined with radioactive MIBG, can give long-lasting palliation [4,5]. Local treatment of the liver, however, remains favorable due to reduction of the tumor load. The metastases are usually not amenable for resection, but HAE and the newer technique of RFA might be effective in the treatment of these patients.

Results of local treatment of this tumor are specifically interesting because of its histological characteristics. Arterial hypervasculation of liver metastases from carcinoid tumors argues in favor of hepatic arterial embolization. In addition, it is thought that RFA might have a specific effect on carcinoid tumors, because of the increased conduction of frictional heating in the hypervascular tissue, resulting in more extensive coagulative necrosis.

Results of HAE and chemoembolization in earlier reports are variable [8,16-19]. Symptomatic improvement after HAE is reported to occur in 64% to 90% [17-19]. Reports on chemoembolization show a slightly better biochemical and tumor response [9,16]. In our series, however, a symptomatic improvement and a corresponding objective biochemical response are only seen in 3 of 13 (23%) patients treated with HAE. The median decrease of urinary 5-HIAA excretion was 32%. It can be hypothesized that HAE was applied to late in the clinical course. The optimal moment of embolization in the clinical course has been discussed in a Swedish study of 29 patients with carcinoid syndrome, but early embolization appeared to be as effective as late embolization [8]. In addition, side effects in our series were impressive leading to a protracted recovery in several cases. It must be noted that all patients had a considerable duration of symptoms before treatment (median 22; range 8 – 193) and systemic therapy was failing. Remarkably, the 3 patients that did benefit from this treatment had a relatively short duration of symptoms (10, 11 and 16 months). This suggests that embolization in an earlier stage might be more effective.

The question is if the same holds true for RFA. RFA might offer a more simple local treatment compared to HAE. The good local tumor control with a satisfactory duration of effect on symptoms has been suggested in several reports of carcinoid patients [12-15]. Although the experience is limited to small series, this may indicate that an early intervention with RFA is favorable.

In our report the three patients treated with RFA showed an impressive biochemical response. RFA could ameliorate symptoms in 2 of three patients with duration of over 13 and 25 respectively. Although the measured 5-HIAA values before treatment in these 2 patients were relatively low, they suffered from incapacitating symptoms at this point and a considerable decrease in 5-HIAA values as well as improvement of symptoms could be reached after RFA. Complications are reported to be minimal and include hepatic failure, hepatic abscess and hepatic infarction [10,12]. In our report one case of transient cholestasis occurred after RFA. In our series RFA is a safe and, when performed percutaneously, a minimally invasive procedure which may provide good local tumor control. Although these results are only first experiences, the excellent biochemical response and duration of symptomatic improvement encourage considering this technique as a treatment in cases of metastatic carcinoid syndrome.

Difficulty in evaluating local ablation methods lies in the fact that patients with advanced stage carcinoid syndrome all have specific characteristics, e.g. duration and degree of symptoms, localization of hepatic metastases and site of the primary tumor. To further explore the possibilities and indications of local treatment options all these factors should be taken account for in future studies.

## Conclusion

We find these results encouraging to broaden the indication of treatment with RFA and to reconsider the time of treatment with embolization. The treatment of carcinoid syndrome remains a challenge for the physician and all therapeutic options must be considered carefully.

## Competing interests

The author(s) declare that they have no competing interests.

## Authors' contributions

**VM **carried out the assembly and analysis of the data and drafted the manuscript.

**JZ **and **RH **participated in the design of the study and provided study material and patients.

**RK **and **WP **were responsible for the hepatic artery embolizations and percutaneous RFA procedure and collected all the radiological data.

**FC **carried out the intra-operative RFA procedures and conceived of the study.

**BT **participated in the concept and design and coordination of the study.

All authors read and approved final version for publication

**Figure 1:**
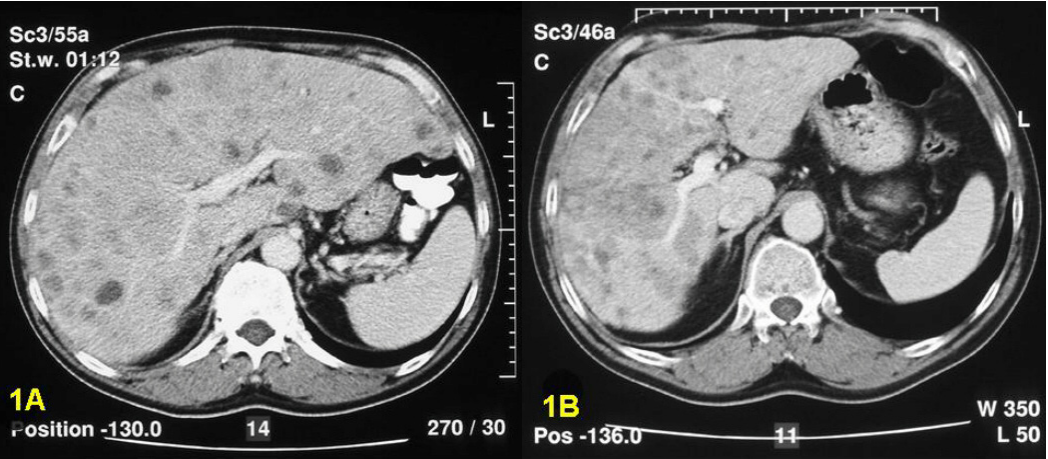
In patient 12 the initial CT-scan (figure 1A) of the liver shows multiple small nodules in both liver lobes. After HAE (figure 1B) the metastases are confluent and the total volume of the liver has decreased remarkably. Symptoms improved and a biochemical response was obvious.

**Figure 2:**
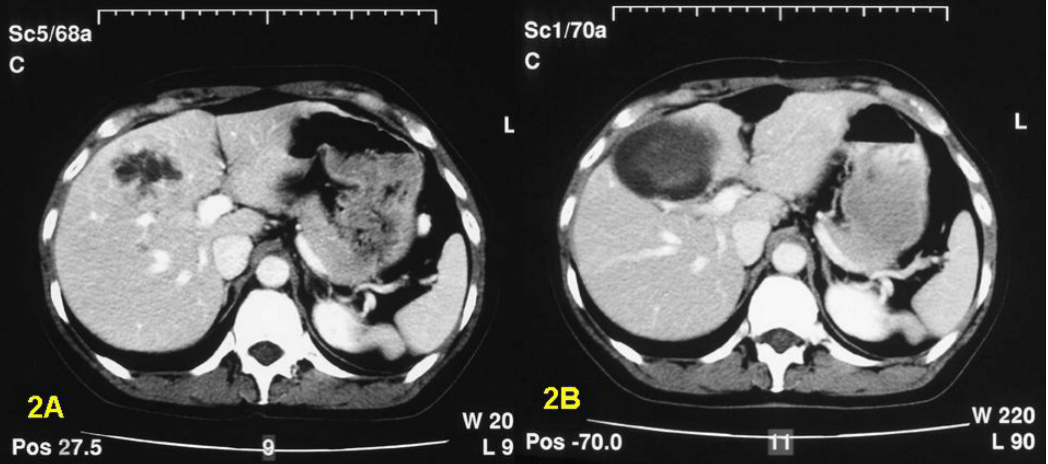
This slide of the CT-scan of patient 14 shows one dominant metastasis in the right liver lobe, but several other small nodules are present (figure 2A). After RFA necrosis with typical cystic appearance developed (figure 2B).

**Figure 3:**
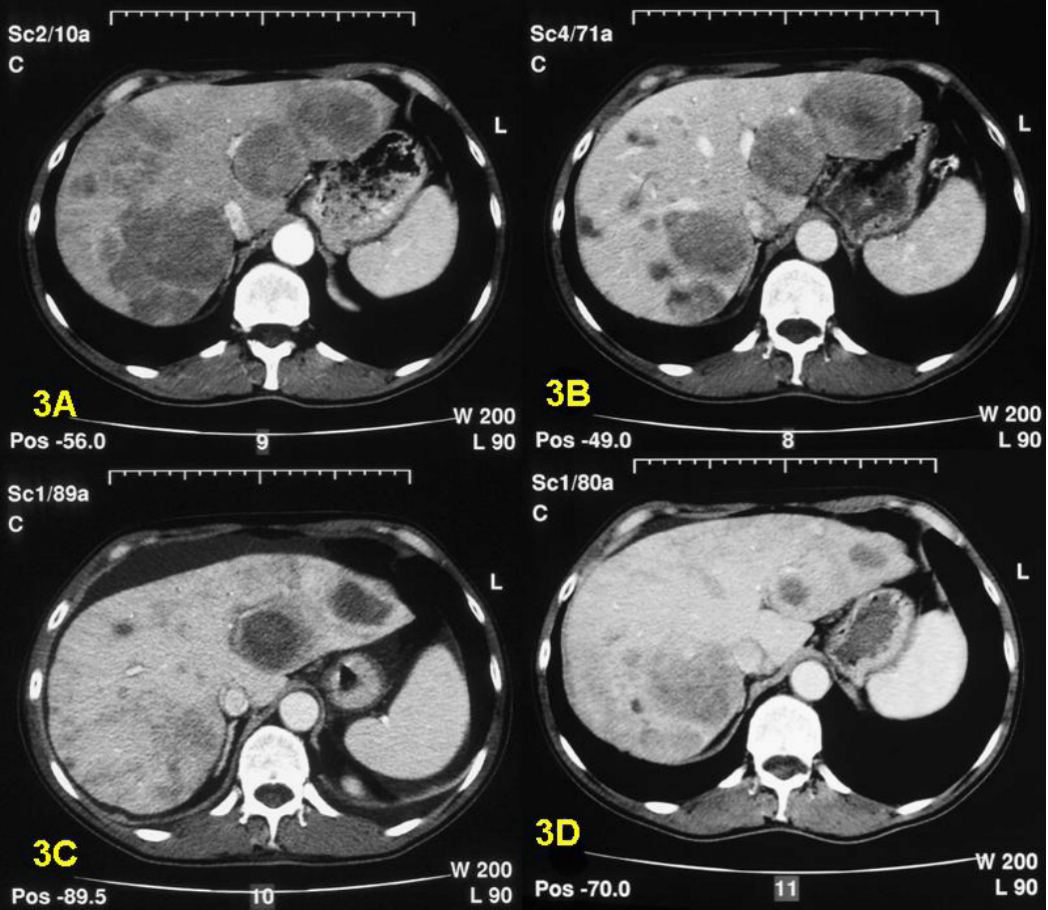
CT-scan of patient 13 reveals several small and larger metastases in both liver lobes (figure 3A). HAE of the right liver lobe resulted in fair tumor reduction (figure 3B). Two large metastases in the left liver lober were treated by RFA and show characteristic cystic appearance (figure 3C). The end result six months after the RFA treatment shows a significant decrease of tumor mass in both liver lobes (figure 3D).
